# 

**Published:** 1890-09

**Authors:** 


### CONTENTS.

*EDITORIALS.* PAGE

The Treatment of Affections of the Heart, . . . . . . . 385
Chronic or Interstitial Pneumonia, . . . . . . . . 387
Dr. P. P. Wells, . . . . . . . . . . . . . . 388
Destroying the Bacillus of Tubercle, . . . . . . . . . 388

*MISCELLANEOUS.*

The Importance of Choosing the Indicated Remedy in the Minimum Dose. F. C. Hood, M. D., . . . . . . . . . 389
Verified Provings of Oxygen. S. Swan, M. D., . . . . . . 397
A Clinical Case. W. A. Yingling, M. D., . . . . . . . 400
Cases from the Practice of Dr. Hansen. S. L., . . . . . . 401
Hermaphroditism Complicated with Extrophy of Bladder. Rita Dunlevy, M. D., . . . . . . . . . . . . . 405
A Funny Symptom, Sulphur 55M. M. A. A. Wolff, M. D., . . 407
Proceedings of The International Hahnemannian Association. J. W. Thomson, . . . . . . . . . . . . . 410
Therapeutics of the Throat. E. B. Nash, M. D., . . . . . 410
Dr. Preston's Case in the August No. W. S. Gee, M. D., . . . 413
Another Criticism of Dr. Preston's Case. Clarence N. Payne, M. D., 414
British Medicinal Plants. Alfred Heath, M. D., . . . . . 415
The Provings of Natrum Muriaticum. A. McNeil, M. D., . . 418
Help Wanted and Received. Edward T. Balch, M. D., . . . 419
A Carbolic Acid Proving. C. M. Selfridge, M. D., . . . . . 420
A Proving of Lachesis $^{200}$. S. Mills Fowler, M. D., . . . . . 421
Dr. T. F. Allen on Homœopathy. E. W. Berridge, M. D., . . 421
Errors in Dr. T. F. Allen's Encyclopedia and Index. E. W. Berridge, M. D., . . . . . . . . . . . . . 425
Sanicula Spring Water. J. G. Gundlach, M. D., . . . . . 426
Antiseptic Dressing. G. M. Pease, M. D., . . . . . . . 427

*BOOK NOTICES.*

Recollections of General Grant. By George W. Childs, . . . 429
Diseases of the Eye and Ear. By C. H. Vilas, M. D., . . . 430
Practical Sanitary and Economic Cooking. By Mrs. Mary Hinman Abel, . . . . . . . . . . . . . . 430
Census Bulletin No. 7, . . . . . . . . . . . . 430
How to Preserve Health. By Louis Barkan, M. D., . . . . 431
The Neuroses of the Genito-urinary System. By Dr. R. Ultzmann, 431
Practical Electricity in Medicine and Surgery. By Drs. Liebig and Rohé, . . . . . . . . . . . . . . 431
The Polytechnic, . . . . . . . . . . . . . 432

*NOTES AND NOTICES.*

Removals, . . . . . . . . . . . . . . 432

